# The prevalence and potential fisheries consequences of *Heterosporis sutherlandae* in a Minnesota lake

**DOI:** 10.1371/journal.pone.0199580

**Published:** 2018-06-25

**Authors:** Megan M. Tomamichel, Nathaniel C. Hodgins, Paul A. Venturelli, Nicholas B. D. Phelps

**Affiliations:** 1 Department of Fisheries, Wildlife, and Conservation Biology, University of Minnesota, St. Paul, Minnesota, United States of America; 2 Minnesota Aquatic Invasive Species Research Center, University of Minnesota, St. Paul, Minnesota, United States of America; 3 Windom Area Fisheries Office, Minnesota Department of Natural Resources, Windom, Minnesota, United States of America; 4 Department of Biology, Ball State University, Muncie, Indiana, United States of America; Texas A&M University, UNITED STATES

## Abstract

*Heterosporis sutherlandae* is an emerging microsporidian fish parasite in the Great Lakes region. *H*. *sutherlandae* forms lesions in the muscle tissue of fishes important to aquaculture and sport fishing. These lesions render the filet inedible and may have fitness consequences. We evaluated the prevalence and severity of *H*. *sutherlandae* among yellow perch (*Perca flavescens*) in a known-positive Minnesota lake, and used an equilibrium yield model to evaluate impacts on harvest. Twenty-eight percent of the 400 yellow perch sampled were infected with *H*. *sutherlandae*. Males were 1.5 times more likely to be infected than females and were more severely infected. The presence of the parasite did not vary with relative weight or age, but infection severity was highest among older individuals that were in better condition. These results suggest that males are more susceptible to infection, and that infection is not associated with maturity or a gape-limiting food source. These results also suggest that heterosporosis increases in severity with time or by increased exposure. Our equilibrium yield model found that a 10% increase in mortality due to *H*. *sutherlandae* could result in 30% and 10% reductions in yield and mean catch weight, respectively. The results of this study direct future field sampling and laboratory experiments to further understand and predict the impacts of this parasite.

## Introduction

*Heterosporis sutherlandae* was first confirmed in 2000 by Sutherland et al. [1] in yellow perch (*Perca flavescens*) from WI and MN, respectively, and causes the disease heterosporosis. This microsporidian parasite has since been reported in 26 waterbodies in Minnesota, 16 in Wisconsin, two in Michigan and one in Ontario, and has been identified as a disease of concern by the Great Lakes Fishery Commission [2]. In addition to yellow perch, susceptible fishes include recreationally and ecologically valuable species such as walleye (*Sander vitreus*), northern pike (*Esox lucius*), rainbow trout (*Oncorhynchus mykiss*), koi (*Cyprinus carpio*), and baitfish [3].

Members of the genus *Heterosporis* enter the host through the consumption of spores from infected prey or directly from the water column and infect the skeletal muscles of fish hosts [2, 4–7]. As the infection progresses, spores form intracellular sporphorous vesicles that rupture to release additional spores into the surrounding tissue. This process destroys the muscle cells, which can be entirely replaced by mature spores and connective tissue. The result is a concave appearance of the host, and a fillet that appears white or freezer-burned and has a soft/mushy texture [2,8]. Consequently, *H*. *sutherlandae*-infected fish fillets are considered unfit for human consumption.

Microsporidian species have been implicated in increased mortality in both wild and laboratory fish and are recognized as the most common parasite in laboratory zebrafish (*Danio rerio*) [9]. *Glugea hertwigi* was correlated with a mass mortality event of rainbow smelt (*Osmerus mordax*)[10]. *Loma* sp. have caused a 10% increase in mortality in wild juvenile Chinook salmon *Oncorhynchus tshawytscha* [11] and *Enterocytozoon salmonis* increased the mortality of experimentally infected fish by 90% [12]. Phelps et al. [2] suggested that *H*. *sutherlandae*-infected fish may succumb to indirect parasite induced mortality. It is important to understand the prevalence and potential population-level consequences of *H*. *sutherlandae* to inform evidence-based management (e.g. the implementation of prevention and control measures).

In this study, we estimated the prevalence and severity of *H*. *sutherlandae* in yellow perch from Leech Lake (Cass County, MN) as a function of gender, maturity, and size. We also used an equilibrium-yield model to estimate the impact on yellow perch yield, yield per recruit and mean weight of catch. This model assumed a range of elevated mortality rates due to infection, which could be driven by impacts on prey capture or predator avoidance [3], or increased stress [13] leading to increased metabolism [14] and/or secondary infections [15].

## Methods

### Ethics statement

Most samples taken for this study were obtained from annual gill net surveys performed by the Minnesota Department of Natural Resources following their standard operating protocols. Fish were provided to the researchers after samples had been frozen. No formal waiver of ethical approval was obtained for these fish. Bag seining was conducted separately from the Department of Natural Resource’s annual gill net survey. Bag seining was conducted under the conditions of a collection permit reviewed and issued by the Minnesota Department of Natural Resources. Fish were only taken as samples if they had died because of the seine haul, live fish were returned to the lake.

### *H*. *sutherlandae* infection prevalence and severity

We collected yellow perch from Leech Lake (47.1487° N, 94.4207° W) to determine the prevalence and severity of heterosporosis. Leech Lake is a 451 km^2^ glacial kettle lake in northern Minnesota that has been locally known to be infested with *H*. *sutherlandae* since 1990, although not confirmed until 2000 [2]. Sampling gear included gill nets (76.20 m by 1.83 m with 5–15.24 meter panels of bar mesh ranging from 19mm to 51mm) that were set overnight as part of a Minnesota Department of Natural Resources fisheries survey (7–16 September 2004) and a 30.48 m shoreline bag seine (25 September and 3 October 2004). Only yellow perch that died due to acute trauma in the bag seines were examined for *H*. *sutherlandae*; live fish were released.

**Fig 1:**
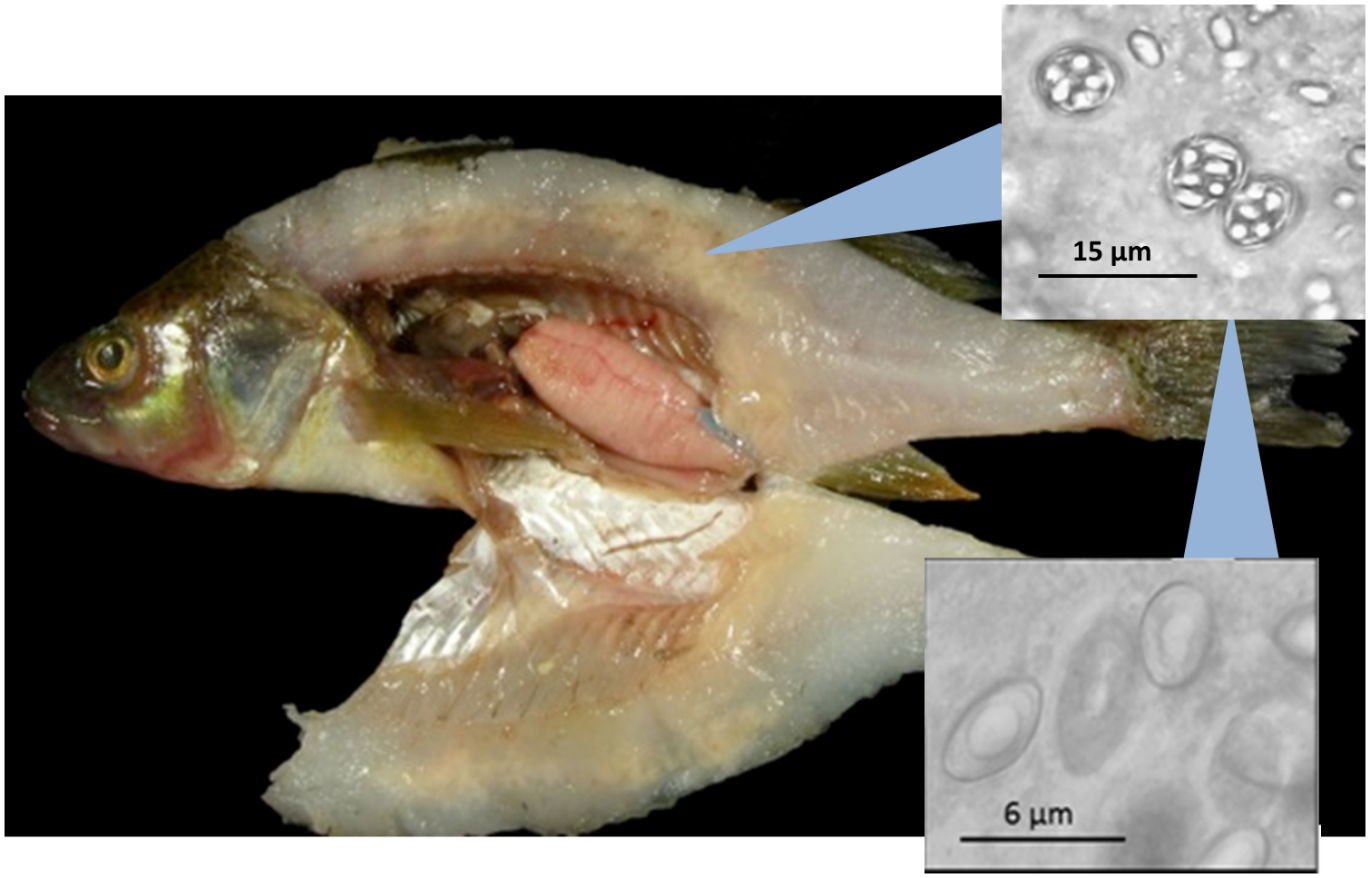
Sampled yellow perch with characteristic lesions of heterosporosis in the muscle tissue. Reprinted from Escobar, et al. (2018) [19] under a CC BY license, with permission from Taylor & Francis Group, original copywrite 2018.

Each fish was weighed to the nearest 0.1 gram, measured for total length in millimeters, and necropsied. We determined sex and maturity via visual inspection of the gonads, and ages were estimated using whole otoliths following Morales-Nin [16]. Filets were visually inspected for soft/mushy, porcelain-white muscle tissue that is characteristic of *H*. *sutherlandae* infections (Fig 1). We removed either a small piece of muscle just above the posterior region of the rib cage or from tissue apparently infected with *H*. *sutherlandae*. We sampled the posterior region of the rib cage for unapparent infections both to provide a uniform sampling location and to target an area known for succumbing to early infection [3]. The sampled tissue was inspected by light microscopy for *H*. *sutherlandae* spores via wet mount at 100x and 400x for up to 2 minutes [17–18]. Infection prevalence is defined as the presence or absence of spores in the wet mount, and infection severity was categorized as none (0 spores/2 minutes of inspection), light (<10 spores/ 2 min), moderate (10–100 spores/ 2 min), or heavy (>100 spores/ 2 min).

**Fig 2:**
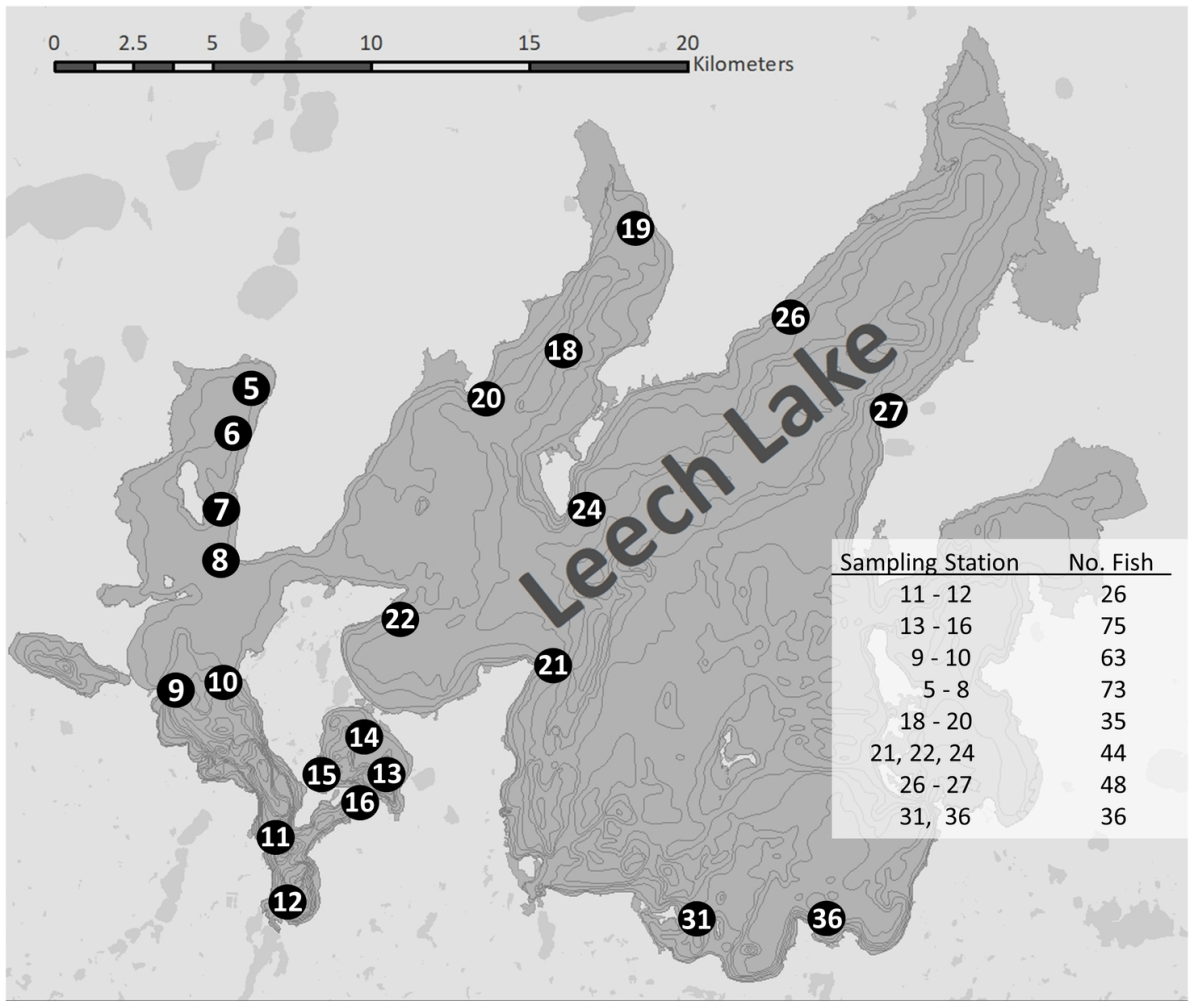
Netting locations on Leech Lake, MN. The numbered dots refer to sampling locations and are referenced in column C of the S1 Table. The column labeled “Sampling Station” in the table indicates which sampling locations were included within one spatial group. The column labeled “No. Fish” indicates the number of fish sampled in each spatial group. Spatial groups were used to examine correlation between sampling locations and *H*. *sutherlandae* prevalence and severity.

We performed various statistical tests to deduce any relationship between *H*. *sutherlandae* prevalence and severity using R (Version 3.4.1) [20]. We calculated the relative weight of each yellow perch from the measured length and weight using the standard weight equation in Willis et al. [21]. To evaluate spatial variation in both prevalence and severity of infection, we grouped sample sites based on geographic proximity prior to evaluation with a generalized linear model and simple linear model with normal error distributions, respectively (Fig 2). Severity of infection related to age, relative weight and location were analyzed via three separate ANOVA with infection severity as the explanatory variable. We coded these models in a Bayesian framework using JAGS [22] and jagsUI [23] in R (4 MCMC chains, 100,000 iterations, 50,000 burn-in iterations, thin rate = 10, uninformative priors on all parameters). A Bayesian approach allowed us to generate pairwise comparisons among groups (via derived parameters) and assess significance without post-hoc analysis [24]. Prevalence of infection related to age, relative weight and location was analyzed with separate logistic regression models with Bernoulli error distributions. We performed a chi-squared test to evaluate the prevalence and severity of infection per sex and the equivalence of the sex ratio of sampled fish.

### Effects of *H*. *sutherlandae-*induced mortality

We used the Jones modification of the Beverton Holt equilibrium yield model [25] (Table 1) to estimate the impact of potential *H*. *sutherlandae* mortality on yield per 1000 individuals, yield per recruit, and the mean weight of 1000 individuals at different rates of instantaneous annual fishing mortality (*F*, range 0.01–1). Typical instantaneous annual recreational fishing mortality (*F*) over all of the length classes in Leech Lake was estimated at 0.5 via analysis of creel data [26]. The Jones modification of the Beverton Holt equilibrium yield model is appropriate when the exponent for the weight:length relationship does not differ from 3 [27], and does not assume that annual length increments remain constant over the range of commercial sizes [25]. The parameters used to model the population of Yellow perch were calculated from this study or Haukos (1995) (Table 1), except for the hypothetical values of additional mortality due to *H*. *sutherlandae* (*H*, range 0–0.2). Yield (*Y*) was calculated using Eq (1) where parameters are defined and quantified in Table 1.

$$Y=\left(F\cdot \;N_{R}\cdot \;exp^{\left(M+F+H\right)(tr\;–\;to)}\cdot W_{\infty }\right)/K)\;\cdot \;\left[\beta \left(X,P,Q\right)\right]$$ (1)

Yield per recruit (*YPR*) and mean weight of catch were calculated for males and females separately using Eq (1), and additional parameters and their values as given in Table 1. (*YPR*) was calculated using (*YPR*) = *Y* / *N*_*R*_ and mean weight of catch ($\overset{-}{W}$) was calculated using $(\;\bar{W})=\;\left(Y/F\right)/(N_{R}/\;\left(M+F+H\right)$.

**Table 1:** Definitions and values of parameters used to predict yield, yield per recruit and mean weight of catch via the Jones modification of the Beverton-Holt equilibrium yield model.

	Parameter	Value	Source
*F*	instantaneous annual fishing mortality rate	0–1 in increments of 0.01	simulated
*N*_*R*_	*N*_*o*_ · *e*^(-*M*. (tr—to))^, number of fish recruited to the population	calculated by model	[28]
*N*_*o*_	original number of fish	1000 individuals	simulated
*M*	instantaneous annual natural mortality rate, given by 10^(-0.0066–0.279log^_10_^(*L*^_∞_^) + 0.6543log^_10_^(*K*) + 0.4634log^_10_^(*T*))^	0.1097074	[29]
*H*	instantaneous annual mortality rate due to Heterosporosis	0–0.2 in increments of 0.05	simulated
*L*_∞_	asymptotic length	339 mm (Female) 268 mm (Male)	derived from von Bertalanffy 1938 equation [30]
*K*	Von Bertalanffy growth coefficient	0.14 (Female) 0.18 (Male)	derived from von Bertalanffy 1938 equation [30]
*T*	mean annual surface temperature of Leech Lake, MN	4.7°C	this study
*tr*	age of recruitment to the fishery	3.3 (Female) 3.5 (Male)	[25, 30]
*t*_*o*_	hypothetical age at which the fish length would be 0 mm	-0.14 (Female) -0.41 (Male)	von Bertalanffy 1938 [30]
*W*_∞_	asymptotic weight	483 g (Female) 230 g (Male)	derived from L_∞_ with length weight relationship [30]
*b*	slope of the length weight relationship	3.3202 (Female) 3.201 (Male)	[25]
β	incomplete beta function	8.336 e-05	[25]
*X*	*e*^-K(*tr*–*to*)^;	0.494702	[25]
*P*	(*M*+*H*+*F*)/*K*	calculated by model	[25]
*Q*	slope of the length weight relationship + 1	4.3202	[25]

## Results

### *H*. *sutherlandae* infection prevalence and severity

We examined 400 yellow perch from Leech Lake for *H*. *sutherlandae* (Fig 2). Total lengths ranged from 38–300 mm (mean = 144.62 mm, SD = 65.4 mm), weight ranged from 0.7–383.3 g (mean = 61.9 g, SD = 69.4 g), and age ranged from 0–7 years (mean = 3.4 years, SD = 2.3 years). All fish identified as positive with visual inspection also tested positive under microscopic inspection. Of the sampled fish, 107 were males, 189 females, and sex could not be determined for 104 individuals. The ratio of 1.76 females for every 1 male was statistically significant (χ^2^(1, N = 296) = 22.7, *P* = 1.9 e ^-6^). The visual and microscopic inspection of muscle from these fish indicated 15% and 28% prevalence, respectively. Males had higher infection rate than females (38% males vs 25% females χ^2^(1, N = 296) = 4.57, *P* = 0.03) and were more likely to be severely infected (χ^2^(3, N = 296) = 12.61, *P* = 0.005). We did not find evidence of correlations between sampling location and either *H*. *sutherlandae* prevalence (*P* = 0.74) or severity (F_7,392_ = 0.41, *P* >0.1) (data in S1 Table).

Prevalence did not correlate with relative weight (*P* = 0.19) or age (*P* = 0.26)_._ However, highly-infected individuals had, on average, 1.48 times higher relative weight (F_1,396_ = 6.153, *P* = 0.0001) and were 1.52 times older than non-infected individuals (F_3, 396_ = 6.069, *P* = 0.0001). Moderately-infected fish did not differ from non-infected fish in terms of relative weight (F_1,396_ = 6.153, *P =* 0.78) or age (F_3, 396_ *=* 6.069, *P* = 0.79). Lightly-infected fish also did not differ from non-infected fish in terms of relative weight (F_1,396_ = 6.153, *P* = 0.095) or age (F_3, 396_ *=* 6.069, *P* = 0.17).

### Effects of *H*. *sutherlandae*-induced mortality

**Fig 3:**
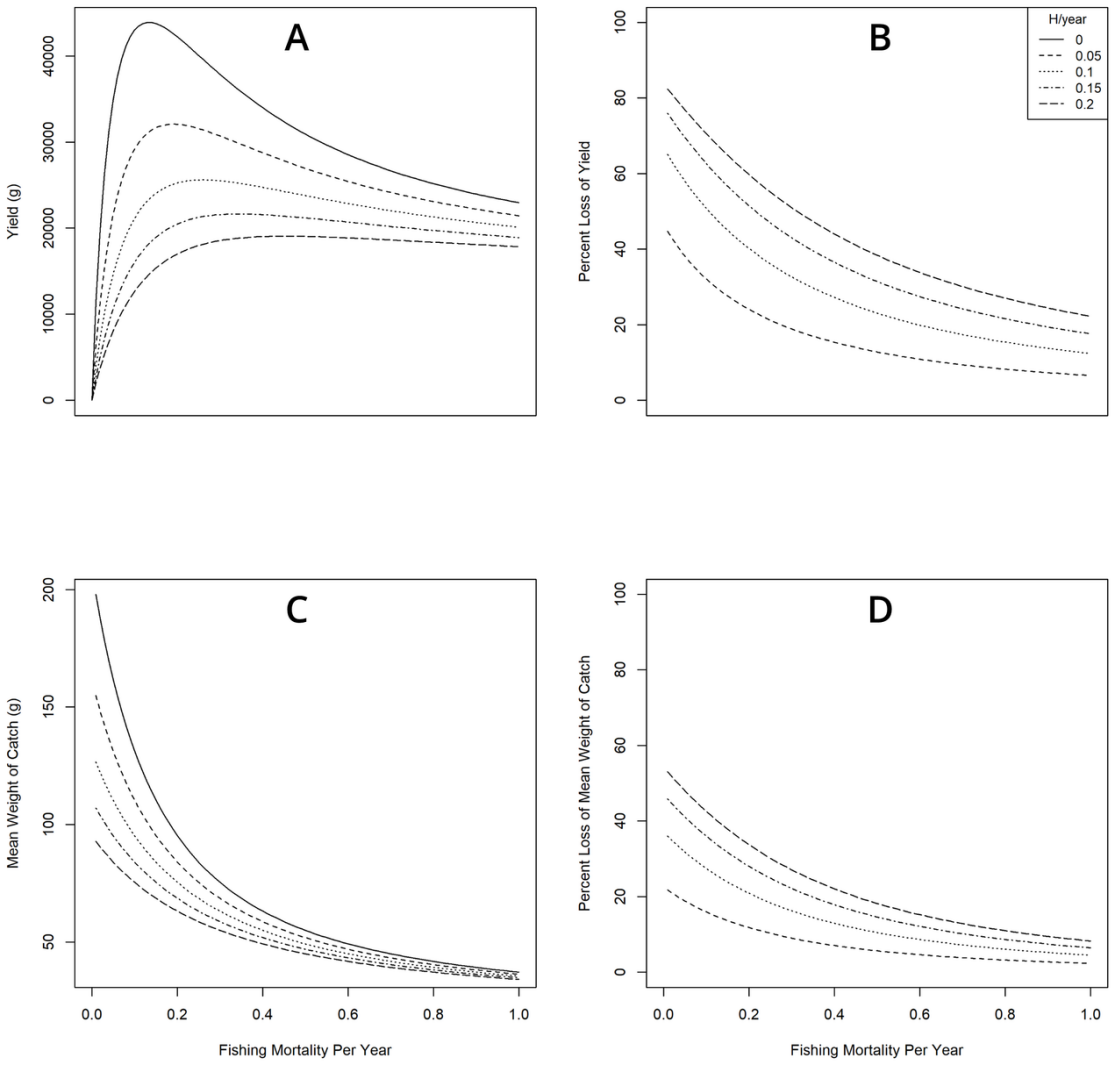
Results of Beverton-Holt equilibrium yield model. (A) Yield and (C) mean weight of catch projections using the Jones modification of the Beverton-Holt equilibrium yield model as a function of fishing mortality for female yellow perch. (B) Percent loss of yield and (D) mean weight of catch as compared to no additional instantaneous annual mortality due to heterosporosis (*H*). Note in panels (B) and (D) *H*/year = 0 is not displayed, because there is zero loss of yield due to heterosporosis.

Our yield-per-recruit model predicted a decrease in the yield and mean weight of catch for female yellow perch with increasing levels of *H*. *sutherlandae* induced mortality over fishing mortality (Fig 3A and 3C). The model predicted a similar relationship for males (S1 Fig). Percent loss of yield and mean weight of catch ranged from 30–90% with fishing mortality (Fig 3B and 3D). The model produced an identical trend for yield per recruit for both males and females. At a 10% increase in mortality due to *H*. *sutherlandae*, the model predicted losses of 30% and 10% in yield and mean weight of catch, respectively (Fig 3A and 3C). Percent loss increased with increasing levels of infection, and decreased with increasing levels of fishing mortality (Fig 3B and 3D).

## Discussion

Our results provide important insight into heterosporosis and suggest that heterosporosis is a disease of concern for yellow perch and perhaps other fishes. The lack of a correlation between *H*. *sutherlandae* prevalence and yellow perch age suggests that the stress or physical contact associated with reproduction is not required for infection. If stress or contact was required, prevalence would have been higher among mature fish. Given that small and young yellow perch are not yet piscivorous, these results also suggest that *H*. *sutherlandae* is not spread solely by a gape-limiting food source; yellow perch may contract the disease via spores in the water column, non-fish food sources (e.g. zooplankton) or vertical transmission. The increase in severity with relative weight and age may demonstrate that heterosporosis is a chronic disease. Younger, smaller fish may have been exposed to the parasite, and then the disease worsened as the fish grew. This result may also indicate that the time that a fish is exposed to *H*. *sutherlandae* in the environment plays a role in the severity of infection, in that larger, older fish have had more time to be re-infected by spores, which could worsen the disease. Another explanation is that larger, older fish in good condition can catch and consume fish already infected with *H*. *sutherlandae*, and therefore receive a higher dose of the parasite, again causing a more severe infection. *H*. *sutherlandae* infections are found in equal prevalence and severity across sampling locations, indicating that *H*. *sutherlandae* is not restricted to a few locations, or strongly influenced by the spatial heterogeneity of relevant abiotic or biotic factors.

A skewed sex ratio towards females in our survey could be attributed to *H*. *sutherlandae* infection, but the link is tenuous. Pre-heterosporosis population assessments on Leech Lake have consistently yielded similar results in male to female ratios [30], perhaps because of gear bias or undiagnosed *H*. *sutherlandae*-infection. Gill nets tend to capture fish with smaller heads and larger bodies than the gill net mesh [31], which describes mature female yellow perch more so than males. However, studies of yellow perch sex and age structure show that healthy populations tend to skew male [32–33] and models of other fish species indicate an optimal sex ratio of 1:1 to provide the most recruitment in a lake system [34]. Therefore, an alternative explanation is that the skewed ratio in Leech Lake is indicative of a stressed population [33, 35–36] or that males have higher mortality rates due to *H*. *sutherlandae* infection than females, thus reducing the number of males in the system.

Our yield-per-recruit model predicted relatively large impacts on yield, yield per recruit and mean weight of catch at reasonably low levels of mortality due to heterosporosis (e.g. 0.1/yr). This result is concerning for the Leech Lake yellow perch fishery, perch fisheries in general, and the wide range of fish species that are susceptible to heterosporosis [3]. The extent to which heterosporosis impacts game fish populations depends on the extent to which heterosporosis impacts mortality and reproduction [37]. Although little information exists about *H*. *sutherlandae*, the mortality that is caused by other microsporidians can drive population cycles in insects [38–40]. Increased mortality has also been associated with microsporidian infection in farmed salmonids [41], and there is evidence that microsporidians limit predator avoidance and prey capture [42].

Given that our data were from 2004 and yellow perch in Leech Lake have not collapsed [43–44], it appears that *H*. *sutherlandae* is not causing a significant impact on yellow perch populations in Leech Lake. However, it may be that the population in Leech Lake has acquired a resistance to this microsporidian [45] or was responding to a stressor such as an unusual weather pattern [12], a pollutant [46], increase in transmission due to high population density [47], or a simultaneous disease outbreak [48] that caused a high rate of disease impact and spread [49]. This implies that a heterosporosis outbreak could occur and create a significant loss of harvest, both due to mortality caused by infection and angler discard of infected filets, if the disease were to infect a naïve population, or if a stressor were to return.

Until further studies are performed and more details regarding the impact of heterosporosis on a host population are known, we recommend a conservative management approach to reduce the risk of heterosporosis exposure to naïve populations. Limiting the harvest of wild-caught baitfish from known-positive waters should be considered given the potential for baitfish to carry important diseases, including heterosporosis [3,50]. We also recommend educating the public regarding proper disposal (i.e. freeze infected tissue prior to disposal [3]) and encourage reporting and confirmation of suspect-positive fish to better define the current distribution of *H*. *sutherlandae*.

Given also that microsporidians i) can spread quickly [51] and widely through fish populations [52], ii) are the most common pathogen detected in laboratory zebrafish [9], and iii) can be resistant to routine lab disinfection protocols [53], we recommend research to determine the frequency of and mechanisms that contribute to heterosporosis-induced mortality. Future field and lab work should also investigate pathways of infection, transmission rates, physiological and behavioral effects, and how these vary with demographics and ecology. This information will provide important insight into heterosporosis and is necessary for developing more sophisticated population models that incorporate disease dynamics and bioenergetics to predict impacts and inform research and management.

## Supporting information

S1 Fig Results of Beverton-Holt equilibrium yield model for male yellow perch. (A) Yield and (C) mean weight of catch projections using the Jones modification of the Beverton-Holt equilibrium yield model as a function of fishing mortality for male yellow perch. (B) Percent loss of yield and (D) mean weight of catch as compared to no additional instantaneous annual mortality due to heterosporosis (*H*). Note in panels (B) and (D) *H*/year = 0 is not displayed, because there is zero loss of yield due to heterosporosis.
(TIF)

S1 Table Collection data from Leech Lake yellow perch. This dataset contains all of the field data from the yellow perch collected from Leech Lake. The first column is the number of fish collected. Column B is the date that each fish was collected. Column C refers to location captured (Fig 2). Column H and I are indications of whether a fish contained lesions caused by *H*. *sutherlandae* spores (Y) and/or if spores were detected under the microscope (Y). Column J refers to the classification of infection severity by the number of spores detected under the microscope (none (0 spores/2 minutes of inspection), light (<10 spores/ 2 min), moderate (10–100 spores/ 2 min), or heavy (>100 spores/ 2 min)). Columns K-N reference the presence of different life stages of the *H*. *sutherlandae* parasite (sporophorous vesicles are abbreviated as SPOV). The final two columns (O and P) reference photos of lesions or *H*. *sutherlandae* spores that are not included in this article.
(XLSX)

S1 Text Permission to recreate Fig 1 under creative commons license. (PDF)
