# Supplementary figures and images for: The prevalence and potential fisheries consequences of Heterosporis sutherlandae in a Minnesota lake
Source: PLoS One. 2018 Jun 25;13(6):e0199580. doi: 10.1371/journal.pone.0199580 (PMC6016923; doi:10.1371/journal.pone.0199580)

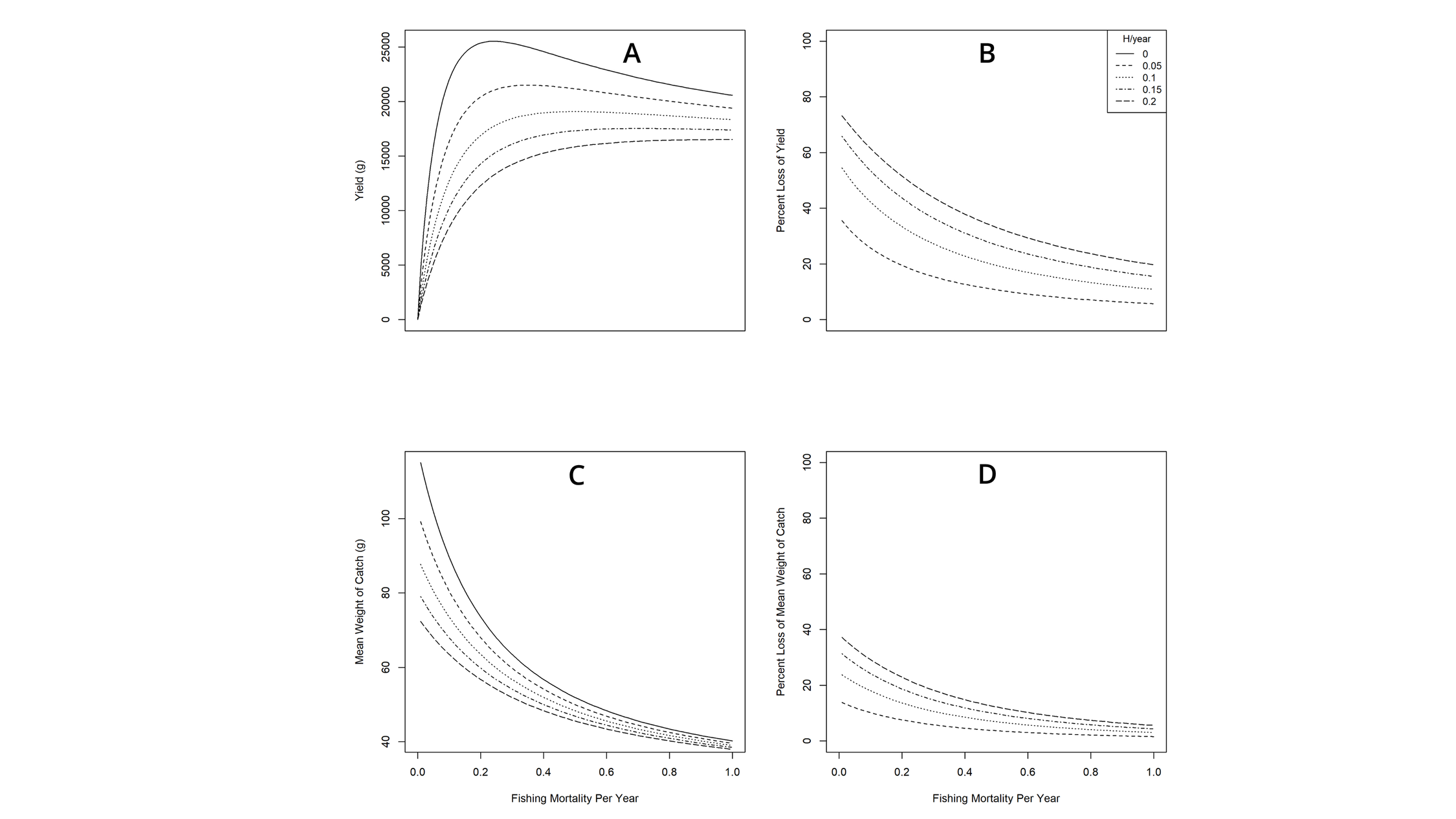

Supplement: S1 Fig — (A) Yield and (C) mean weight of catch projections using the Jones modification of the Beverton-Holt equilibrium yield model as a function of fishing mortality for male yellow perch. (B) Percent loss of yield and (D) mean weight of catch as compared to no additional instantaneous annual mortality due to heterosporosis (H). Note in panels (B) and (D) H/year = 0 is not displayed, because there is zero loss of yield due to heterosporosis. (TIF) [file pone.0199580.s001.tif]
