# Supplementary material for: The prevalence and potential fisheries consequences of Heterosporis sutherlandae in a Minnesota lake
Source: PLoS One. 2018 Jun 25;13(6):e0199580. doi: 10.1371/journal.pone.0199580 (PMC6016923; doi:10.1371/journal.pone.0199580)
Supplement: S1 Text — (PDF) [file pone.0199580.s003.pdf]

Taylor & Francis
Taylor & Francis Group

Our Ref: P051118-06/BRFS

11 May 2018

Dear Megan Tomamichel for PLOS ONE,

Material Requested: Figure 3 in
Luis E. Escobar, Sophie Mallez, Michael McCartney, Christine Lee, Daniel P. Zielinski, Ratna Ghosal, Przemyslaw G. Bajer, Carli Wagner, Becca Nash, Megan Tomamichel, Paul Venturelli, Prince P. Mathai, Adam Kokotovich, Joaquin Escobar-Dodero & Nicholas B. D. Phelps (2017)
Aquatic Invasive Species in the Great Lakes Region: An Overview
***Reviews in Fisheries Science & Aquaculture*, 26 (1): 121-138.**
DOI: 10.1080/23308249.2017.1363715

Thank you for your email requesting permission to reproduce your above material in your future publication in *PLOS ONE*.

As you are the author of the above article, and the main author/editor of the forthcoming publication, we will be pleased to grant non-exclusive world rights in all languages for **print and eBook** usage without charge on the condition that you include a full acknowledgement to the original source of publication and insert a reference to the Journal’s web site: https://www.tandfonline.com

Permission is granted to publish your figure under a CC BY license.

Permission must be sought for any further use.

You will also need to obtain permission from any co-authors of this article.

This permission does not cover any third party copyrighted work which may appear in the material requested.

This license does not cover Taylor & Francis content being republished in a custom publishing programme or database or any individual chapters’ sales.

Thank you very much for your interest in Taylor & Francis publications. Should you have any questions or require further assistance, please feel free to contact me directly.

Sincerely,

Mary Ann Muller

Permissions Coordinator

E-mail: maryann.muller@taylorandfrancis.com

Telephone: 215.606.4334
